# Effect of Loading Rate on Tensile Strength and Fracture Stress of Chocolate

**DOI:** 10.1111/jtxs.70021

**Published:** 2025-04-21

**Authors:** Šárka Nedomová, Vojtěch Kumbár, Jan Trnka, Veronika Šafránková, Renáta Dufková, Jiří Votava, Luděk Hřivna, Jaroslav Buchar

**Affiliations:** ^1^ Department of Food Technology Mendel University in Brno Brno Czech Republic; ^2^ Department of Technology and Automobile Transport (Section Physics) Mendel University in Brno Brno Czech Republic; ^3^ Institute of Thermomechanics, Czech Academy of Science Prague Czech Republic

**Keywords:** Brazilian test, chocolate, fracture properties, loading rate, split Hopkinson pressure bar, tensile strength

## Abstract

The fracture properties of five types of chocolate (dark, extra dark, milk, white, and ruby) were investigated using an indirect tensile test known as the Brazilian test. Two different loading rates of 0.0017 m/s and around 12 m/s were used through the universal testing machine TIRATEST and split Hopkinson pressure bar (SHPB) technique. Results show that the tensile fracture stress increases with the loading rate. The sensitivity of the fracture stress at low‐loading rates is lower than that at the high‐loading rates. The obtained results can be used in industry for the correct processing of chocolate products and their transport. The presented methods can be also used to detect defects in chocolate products.

## Graphical abstract

Five different chocolates (extra dark, dark, milk, white, and ruby) were tested using two approaches (low/high‐loading rate) to determine their mechanical behavior. Records of chocolate deformation from a high‐speed camera were presented. Fracture of specimens starts in the middle of the specimen and tensile fracture stress increases with the loading rate.

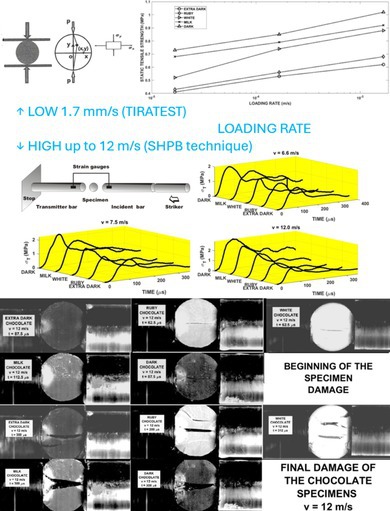

## 1 Introduction

The fracture properties of solid foods, for example, fracture stress and strain, are highly correlated to the textural attributes perceived by humans either through snapping in fingers or during mastication (Chen 2009; Kim et al. 2012). This is valid namely for the chocolate where good snap and hardness are key mechanical features affecting a consumer's perception of quality (Beckett 2008). The fracture stress is affected namely by the chocolate microstructure as reviewed in (Zhao et al. 2018). Instead of this factor, the loading rate (LR) also plays a significant role (Bikos et al. 2021). The fracture of chocolate was studied using the universal testing machines in tension and compression. The three‐point bending test was also used. During this test, the force required to break the specimen results from both pressure and tension. The study of chocolate fracture in tension traction tests was performed on plain chocolate at different levels of freezing by anchoring rectangular sticks of chocolate between two clamps in a traction machine under uniaxial traction (Tremeac et al. 2008). However, as chocolate is brittle, it is difficult to mount between two clamps in the traction machine without inducing stresses, causing it to break or weaken preferentially at the clamp contact points prior to the start of the test. If the fracture behavior is studied by tensile testing, specimens can be glued to some more rigid plates, for example, steel plates, which are fixed in the testing machine.

To overcome these difficulties, the indirect tensile test (Brazilian test) was suggested (Nedomová et al. 2013). The results of this method showed that the tensile strength of chocolate increases with the LR. The LR was limited to the value of 0.0017 m/s.

The aim of this paper was to study the effect of higher LRs on tensile strength and fracture stress of five different types of chocolate. In the given text, the results of low velocity loading of chocolate were noticeably extended to significantly higher loading velocities (up to about 12 m/s). The study of the fracture development was also performed. It has been shown that there are at least two regions that differ in fracture strength sensitivity to the LR. The comparison of the results of both loading techniques and multiple LR regimes (quasi‐static and impact), including the addition of fracture development of five different types of chocolate, proves to be a novelty in this field.

## 2 Material and Experimental Methods

### 2.1 Experimental Samples

Five chocolate masses were used for the experiments—extra dark chocolate, dark chocolate, milk chocolate, white chocolate, and ruby chocolate. Detailed characteristics (technology, detailed composition, and nutritional data) of these chocolate masses are given in Table 1 and some others in the previous studies by Kumbár et al. (2021), (2023).

**TABLE 1:** Nutritional data of the chocolates.

Chocolate	Nutritional information per 100 g					Composition
	Energy value	Fats	Carbohydrates	Proteins	Salt	
		Of which saturated fatty acids	Of which sugars			
EDC	2576.0 kJ	53.0 g	14.6 g	15.0 g	0.2 g	100% cocoa components
		20.0 g	1.0 g			
DC	2264.6 kJ	35.0 g	46.9 g	5.7 g	< 0.005 g	55.5% cocoa components, sugar, cocoa butter (35.0%), emulsifier: soy lecithin (E322), aroma: natural vanilla
		21.6 g	44.0 g			
MC	2297.2 kJ	33.3 g	55.6 g	5.8 g	0.206 g	36.5% cocoa components, sugar, cocoa butter (29.0%), whole milk powder, lactose, whey powder, emulsifier: soy lecithin (E322), aroma: natural vanilla
		20.4 g	54.7 g			
WC	2360.5 kJ	35.1 g	56.1 g	6.4 g	0.234 g	28% cocoa components, sugar, cocoa butter (28%), whole milk powder, emulsifier: soy lecithin (E322), aroma: natural vanilla
		21.3 g	56.1 g			
RC	2356.0 kJ	35.9 g	49.6 g	9.3 g	0.270 g	47.3% cocoa components, sugar, cocoa butter, whole milk powder, emulsifier: soy lecithin (E322), acidity regulator: citric acid, aroma: natural vanilla
		21.5 g	48.5 g			

Abbreviations: DC, dark chocolate; EDC, extra dark chocolate; MC, milk chocolate; RC, ruby chocolate; WC, white chocolate.

The chocolate masses were tempered using a tempering machine Color EX (Selmi Chocolate Machinery, Italy) according to the tempering curves, see Table 2. Tempering temperatures shown in Table 2 are given by the manufacturers of the individual chocolate masses. These are the long‐established key temperature values necessary for correct tempering. The tempered chocolate masses were shaped in a special form (AISI 316 L food‐grade stainless steel), followed by the displacement of air bubbles using vibration (1 min). The frequency and amplitude of the vibrations were constant and given by the manufacturer according to many years of experience. The masses were then cooled to 16°C within 3 min. The scraping off of the excess chocolate from the surface of the form (to ensure a uniform mass of the samples) was followed. After that, cooling continued at 10°C for 45 min. After complete solidification, the samples were tapped from the form and further stored at a temperature of 15°C. Vibration was chosen as the optimum method to remove air bubbles from the sample. Time and temperature of storage of samples prior to the removal of excess chocolate mass were set to produce samples with smooth and regular shapes.

**TABLE 2:** Specific tempering temperatures of chocolates.

Chocolate	Melting temperature (°C)	Cooling temperature (°C)	Working temperature (°C)
EDC	55	28.5–29.5	29.5–30.5
DC	45–50	28–29	31–32
MC	40–45	27–28	30–31
WC	40–42	25–26	28–29
RC	45	27	28.5–29.5

Abbreviations: DC, dark chocolate; EDC, extra dark chocolate; MC, milk chocolate; RC, ruby chocolate; WC, white chocolate.

The various mechanical properties of these chocolates were studied in the previous papers by Nedomová et al. (2023), Kumbár et al. (2023), and Kumbár et al. (2024). In Table 3, some material properties that are used for the discussion of results are presented. The specimens of cylindrical shape (14 mm in diameter and 5 mm in thickness) have been prepared. The thickness of the specimens plays a dominant role in the achieving of stress equilibrium. Generally, it is accepted that the thickness of the specimen should be one half of the specimen diameter, see Chen and Song (2011). For the study of tensile strength and fracture stress propagation is thus use a specimen of lower thickness is thus used; in our works, it should be lower than about 7 mm.

**TABLE 3:** Selected material properties of the tested chocolates (*n* = 5).

Chocolate	Density *ρ* (kg/m^3^)	Young modulus *E* (MPa)	*c*_*L*_ (m/s)	*c*_*T*_ (m/s)
EDC	1170.0	1264.5	1982	689
DC	1269.4	2303.6	2208	798
MC	1263.4	1780.3	2106	700
WC	1252.8	1712.0	2094	649
RC	1244.8	1516.0	2074	611

Abbreviations: *c*_*L*_, velocity of longitudinal elastic wave; *c*_*T*_, velocity of transversal elastic wave; DC, dark chocolate; EDC, extra dark chocolate; MC, milk chocolate; RC, ruby chocolate; WC, white chocolate.

### 2.2 Experimental Technique

The low rates loading of the chocolate specimens was performed using the TIRATEST 27025 testing machine (TIRA Maschinenbau, Germany). For more information on the technique used, see Nedomová et al. (2023).

**FIGURE 1:**
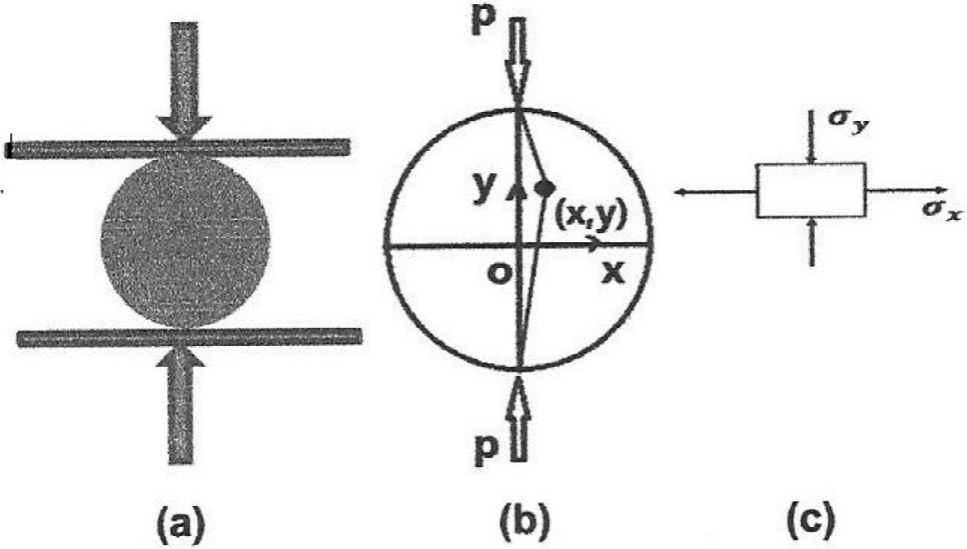
Schematic of the Brazilian test: (a) Brazilian tensile test is performed by applying a concentrated line compressive load across the diameter of a disc shaped specimen; (b) schematic plane view of Brazilian tensile test with Cartesian coordinate; (c) central element of tested specimen, both tensile stress and compressive stress exist simultaneously during a Brazilian tensile test.

The Brazilian/indirect tensile test is performed by applying a concentrated linear compressive load across the diameter of a disc‐shaped specimen, as shown in Figure 1. The loading produces a region of nearly uniform tensile stress perpendicular to the load axis that is sufficient to fracture the specimen. The thickness *L* to diameter *D* ratio range should be from 0.2 to 0.75 (ASTM D3967‐08) in order to achieve a plane stress condition. When the pair forces are applied to a linear elastic Brazilian disc (Figure 1a), the analytical solution of the plane stress, at any given point (*x*, *y*) of the disc (Figure 1b), can be evaluated; see Li and Wong (2013) for details.

**FIGURE 2:**
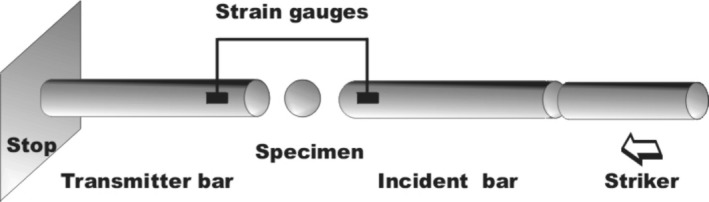
Schematic of the dynamic Brazilian test.

At the center point of the Brazilian disc (Point 0 in Figure 1b), where *x* = *y* = 0, the stress state can be written as:

(1) $$\sigma_{x}=\frac{2P}{\mathrm{\pi LD}}\ldots \text{tension stress}$$

(2) $$\sigma_{y}=-\frac{6P}{\mathrm{\pi LD}}\ldots \text{compression test}$$

(3) $$\tau_{\mathrm{xy}}=0\ldots \text{shear stress}$$

where *P* represents the forces acting on both ends of the specimen. The Equations ((1), (2), (3)) are valid for quasi‐static loading when LRs are limited to about 1 m/min. The loading of the specimen by significantly higher LRs is performed namely using the split Hopkinson pressure bar (SHPB) technique (Czech Academy of Science, Czech Republic) as described by Dai et al. (2010). The SHPB technique used for the study of tensile strength is schematically shown in Figure 2.

This system consists of three main parts:

- First, there is a gas gun enabling the acceleration of the projectile (striker) to some velocity. The gas gun system consists of a high‐pressure reservoir with controls to regulate the pressure of the system. A quick release valve allows the gas to expand and accelerate the striker bar towards the pressure bars. The details of the striker acceleration are described by Rahner et al. (2014).
- The second part is a system of two elastic bars (incident and transmitted).
- The third part is the data acquisition system.

After the impact of the striker on the end of the incident bar, the compressive stress pulse (incident stress pulse) *σ*_*I*_(*t*) is developed. After the impact of this wave on the interface between the incident bar and specimen, some part is reflected back as the reflected stress pulse *σ*_*R*_(*t*) and part is transmitted to the second bar as the stress pulse *σ*_*T*_(*t*). The evaluation of these stress pulses is based on the assumption of the elastic behavior of the bars during the test. The stress pulses are then calculated using the signals from the strain gauges pasted on the bars:

(4a) $$\sigma_{I}=E_{b}\varepsilon_{I}$$

(4b) $$\sigma_{R}=E_{b}\varepsilon_{R}$$

(4c) $$\sigma_{T}=E_{b}\varepsilon_{T}$$

where *E*_*b*_ is the Young's modulus of the bar, *ε*_*I*_, *ε*_*R*_, and *ε*_*T*_ are the incident, reflected, and transmitted strains, respectively.

The incident (loading) stress pulse is characterized by the following parameters:

- The maximum value of the stress (amplitude) *σ*_*Im*_

$$•\;\text{Impulse}\;I_{I}=\int \sigma_{I}\left(t\right)\mathrm{dt}$$

- Energy $w_{I}=\frac{1}{Z_{b}}\int \sigma_{I}^{2}\left(t\right)\mathrm{dt}$, where *Z*_*b*_ denotes the acoustic impedance of the test bar $\sigma_{I}$.

These parameters are then defined also for the remaining stress pulses. The stress pulses enable the calculation of the forces at the interface between the bar and the specimen:

(5a) $$P_{1}\left(t\right)=A_{b}\left[\sigma_{I}\left(t\right)+\sigma_{R}\left(t\right)\right]$$

(5b) $$P_{2}\left(t\right)=A_{b}\sigma_{T}\left(t\right)$$

where *A*_*b*_ is the area of the bars.

The evaluation of the results of the dynamic Brazilian test assumes the stress equilibrium before the specimen failure, that is, *P*_1_ = *P*_2_. Tensile stress in the center of the specimens due to diametral compression is then given by Equation (1) (Wang and Tao 2022):

(6) $$\sigma_{t}=\frac{2A_{b}\sigma_{T}\left(t\right)}{\mathrm{\pi LD}}$$

The dynamic tensile strength *f*_*td*_ is obtained using Equation (1) for the maximum transmitted load. The strain rate follows as (Yu et al. 2004):

(7) $$\dot{\varepsilon }\equiv \frac{\partial \varepsilon }{\partial t}=\frac{1}{E}\frac{\partial \sigma }{\partial t}$$

where ε is the strain, *E* is the elastic modulus of the specimen material, and the derivative is restricted to the linear ramp‐up of the stress history.

Sheikh et al. (2019) suggested the new approach of the LR characterization when the specimen LR is given as the difference of the velocities of the specimen *v*_1_ and bar interfaces *v*_2_:

(8) $$v=v_{1}-v_{2}=\frac{\sigma_{I}-\sigma_{R}}{Z_{b}}-\frac{\sigma_{T}}{Z_{b}}=\frac{\sigma_{I}-\sigma_{R}-\sigma_{T}}{Z_{b}}$$

The knowledge of the LR enables one to evaluate the specimens shortening (displacement) according to:

(9) $$p=\int \mathrm{vdt}=\frac{1}{Z_{b}}\int \left(\sigma_{I}-\sigma_{R}-\sigma_{T}\right)\mathrm{dt}$$

The SHPB technique set‐up consists of two bars of 1000 mm in length and 15 mm in diameter, which are made from poly(methyl methacrylate) material (PMMA). The behavior of the bars during the impact is linear elastic. The acoustic impedance of the bars is *Z*_*b*_ = 2.6486 MPas/m. The striker was made from beech wood in the form of a cylinder of 152 mm in length and 14.8 mm in diameter.

To obtain more information on the specimen behavior, impacts themselves were monitored by a high‐speed camera FASTCAM SA‐Z type 2100K‐M (Photron, Japan) with a maximum frame rate of 2,100,000 fps and a shutter speed of 1.00 μs.

Experiments were performed at the temperature 12°C. At this temperature, specimens of all chocolates exhibited elastic behavior and behaved as a solid body. The contact measurement of specimens' surface temperature after measurement did not show a measurable increase in the temperature.

### 2.3 Statistical Analysis and Software

All the experimental data were analyzed with analysis of variance (ANOVA) and Duncan's test with *p* < 0.05 using the MATLAB statistics toolbox (MathWorks, USA).

## 3 Results and Discussion

### 3.1 Low‐Loading Rates

Loading of the chocolate specimens at low rates corresponding to the quasi‐static loading was performed using TIRATEST. Specimens were compressed as shown in Figure 1 at cross head velocities of 1, 10, and 100 mm/min. In units of m/s, these velocities correspond to 0.0000167, 0.000167, and 0.00167 m/s.

**FIGURE 3:**
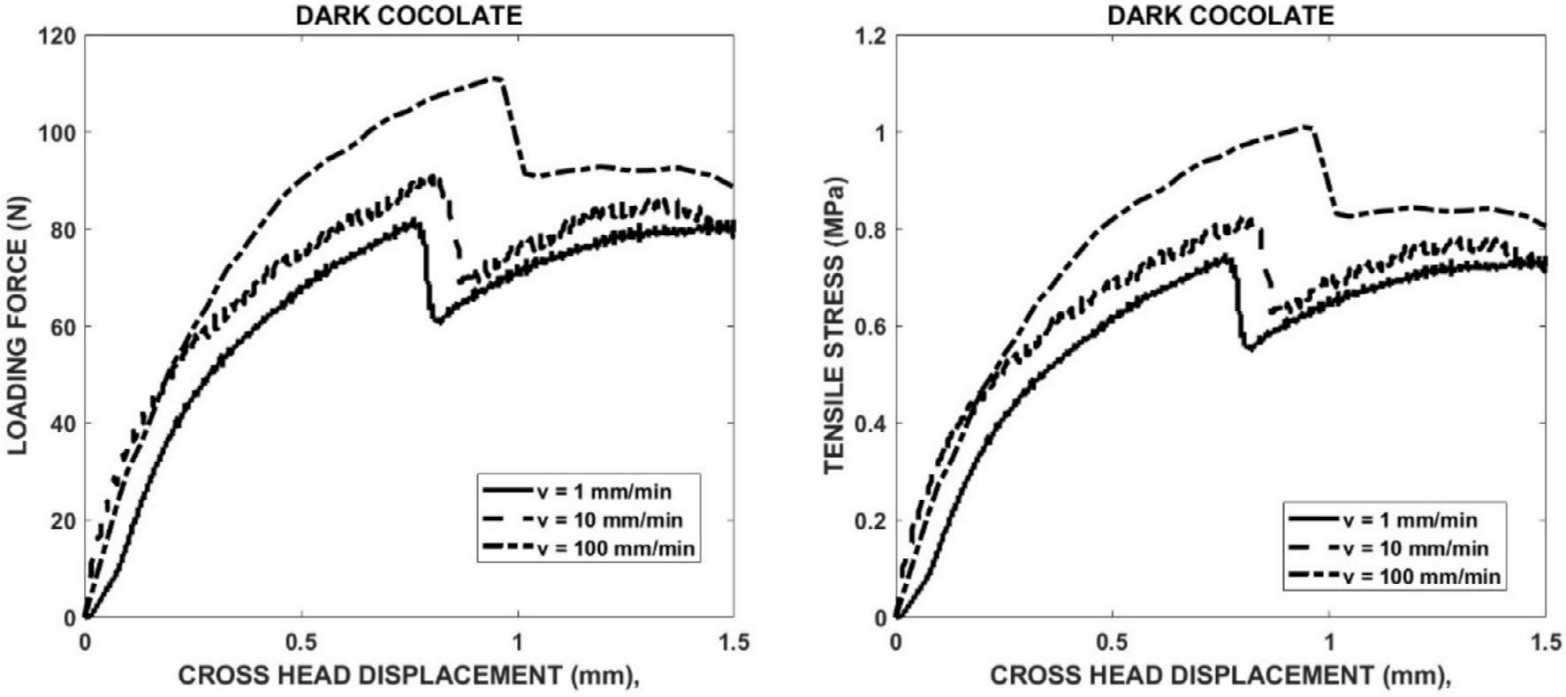
Experimental records of the force–displacement (left part) and tensile stress–displacement (right part).

**FIGURE 4:**
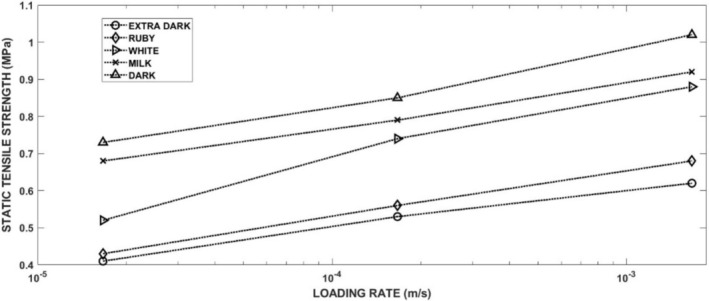
Effect of the loading rate on the tensile strength of different chocolates.

The development of the tensile stress in the specimen leads to the fracture of the specimen. Examples of the experimental records of compressive force versus crosshead displacement are displayed in Figure 3 (left part). All these records are characterized by a drop in the force. The maximum of the force before its sudden decrease corresponds to the moment of the specimen fracture initiation. It has been verified by the experiments where the crosshead displacement was stopped before the force drop. The corresponding tensile stress evaluated using Equation (1) is shown in Figure 3 (right part). This equation is valid up to the specimen fracture. The stress corresponding to the maximum before the drop represents the tensile strength of the tested material. The values of the tensile strength are summarized in Table 4. In this table, the values of the displacement *p* at the fracture are also given. In Figure 4, the tensile strength of the chocolate is plotted as a function of crosshead (loading) rate. It is obvious that the tensile strength increases with the LR. The order of chocolates according to the tensile stress is:

(10) Extra dark→ruby→white→milk→dark

**TABLE 4:** Tensile strength and displacement of the chocolates tested under quasi‐static loading.

Chocolate	Transmitted stress pulse *σ*_*t*_ (MPa)	Displacement *p* (mm)
*v* = 1 mm/min ≡ *v* = 0.0000167 m/s		
EDC	0.41 ± 0.0316^ab^	1.20 ± 0.165126^e^
RC	0.43 ± 0.0804^ab^	0.97 ± 0.066833^cd^
WC	0.52 ± 0.0455^c^	0.91 ± 0.018257^cd^
MC	0.68 ± 0.0258^d^	0.83 ± 0.02582^ab^
DC	0.73 ± 0.0216^e^	0.75 ± 0.018257^ab^
*v* = 10 mm/min ≡ *v* = 0.000167 m/s		
EDC	0.53 ± 0.0365^ab^	1.23 ± 0.045461^e^
RC	0.56 ± 0.042^ab^	1.10 ± 0.049666^d^
WC	0.74 ± 0.0365^cd^	0.98 ± 0.018257^c^
MC	0.79 ± 0.0294^cd^	0.83 ± 0.02582^b^
DC	0.85 ± 0.0392^e^	0.81 ± 0.018257^a^
*v* = 100 mm/min ≡ *v* = 0.00167 m/s		
EDC	0.62 ± 0.0316^ab^	1.28 ± 0.039158^e^
RC	0.68 ± 0.0622^ab^	1.15 ± 0.029439^d^
WC	0.88 ± 0.0294^cd^	1.05 ± 0.024495^c^
MC	0.92 ± 0.0183^cd^	0.95 ± 0.03559^ab^
DC	1.02 ± 0.0548^e^	0.92 ± 0.024495^ab^

*Note:* ^a, b, c, d, e^ different letters in one column means statistically significant differences (*p* < 0.05).

Abbreviations: DC, dark chocolate; EDC, extra dark chocolate; MC, milk chocolate; RC, ruby chocolate; WC, white chocolate.

**FIGURE 5:**
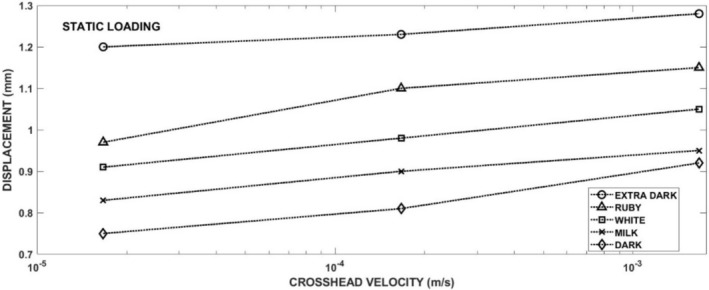
Displacement of the chocolate specimen at its fracture moment.

The values of the fracture strength are lower than those reported during the three‐point bending test (Zhao et al. 2018). This difference may be a consequence of some plastic strain during the Brazilian test. The rate dependence of the tensile strength was also observed in our previous paper (Nedomová et al. 2013). The increase in tensile strength is also a consequence of the increase in the content of cocoa particles. The increase due to the increase in the content of cocoa particles was probably independent of LR. It means that the observed strain rate sensitivity of the tensile strength is probably a consequence of the viscous behavior of the cocoa butter. The increase of the tensile strength with the LR was also observed by Bikos et al. (2021). The specimen displacement (shortening) at the fracture also increases with the LR, as shown in Figure 5.

The value of this displacement increases with the LR. The order of the chocolates according to this parameter is opposite to the order given by Equation (10).

### 3.2 High‐Loading Rates

**FIGURE 6:**
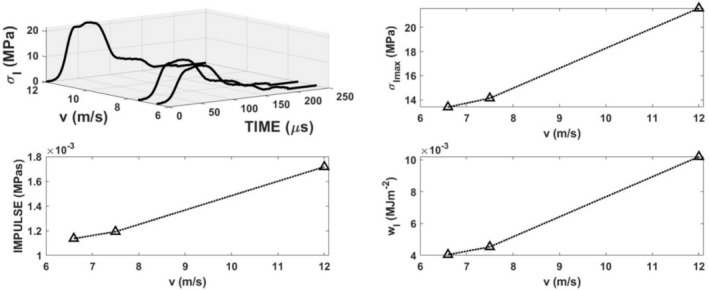
Loading (input) stress pulses recorded for the specimens of the dark chocolate and main pulses parameters as function of the striking velocity *v*.

**FIGURE 7:**
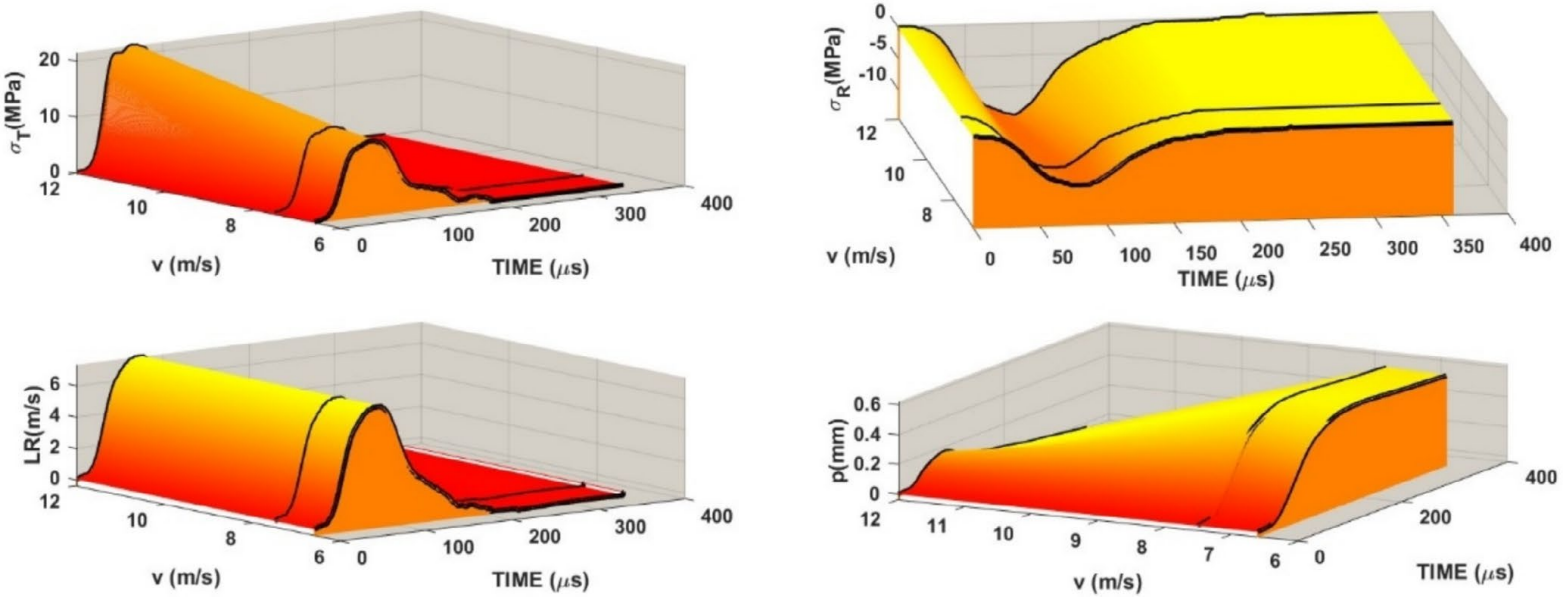
Record of the transmitted and reflected stress pulses recorded for the specimens of dark chocolate. The time history of the loading rate LR using Equation (8) and the specimen displacement *p* (shortening) using Equation (9).

**FIGURE 8:**
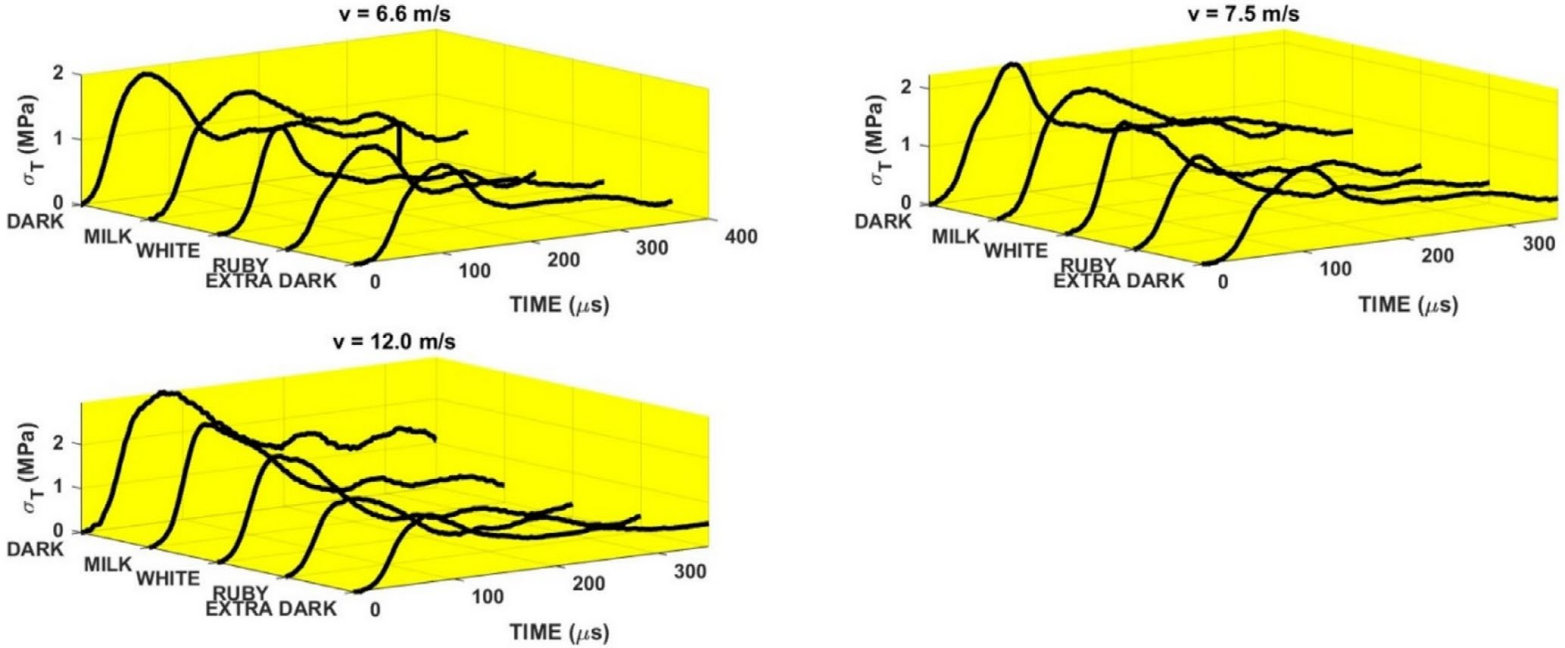
Transmitted stress pulses recorded as a response of chocolate to input stress pulses corresponding to the different striking velocities *v*.

**FIGURE 9:**
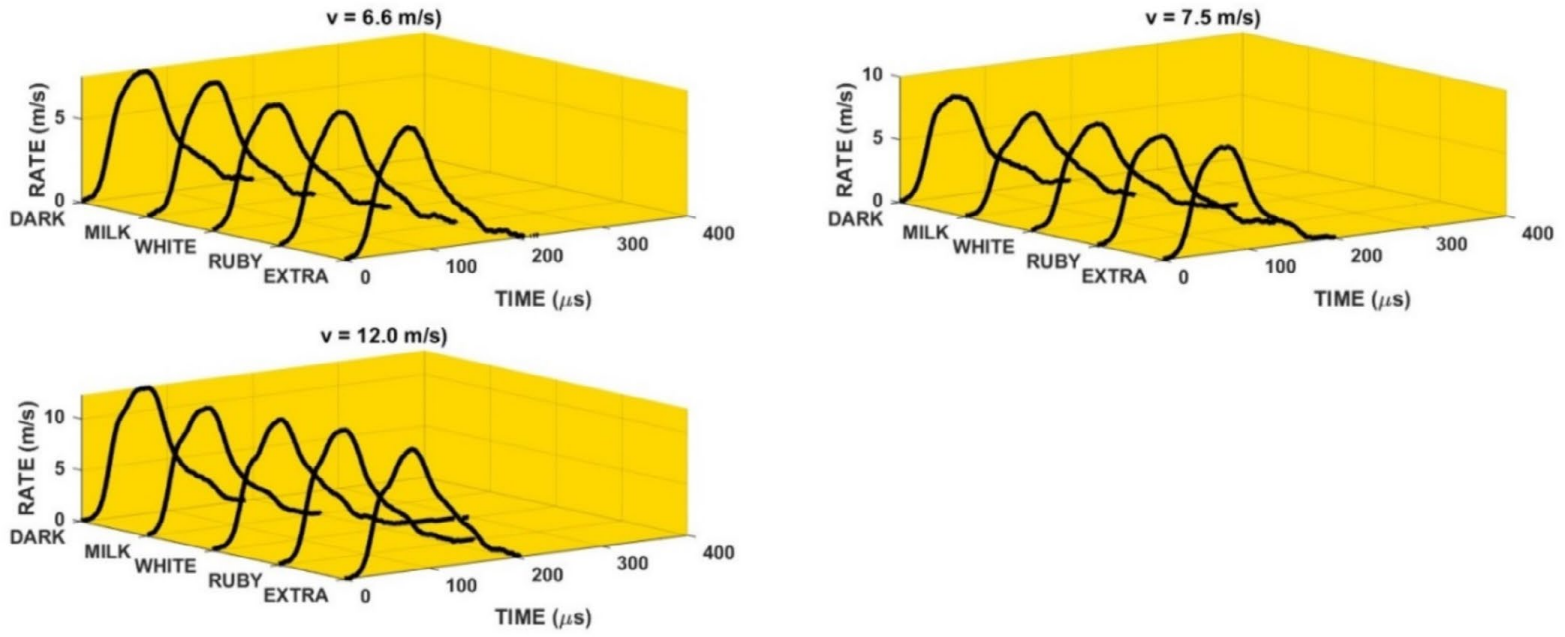
Loading rates (RATE) of the specimens of the different kind of chocolate.

**FIGURE 10:**
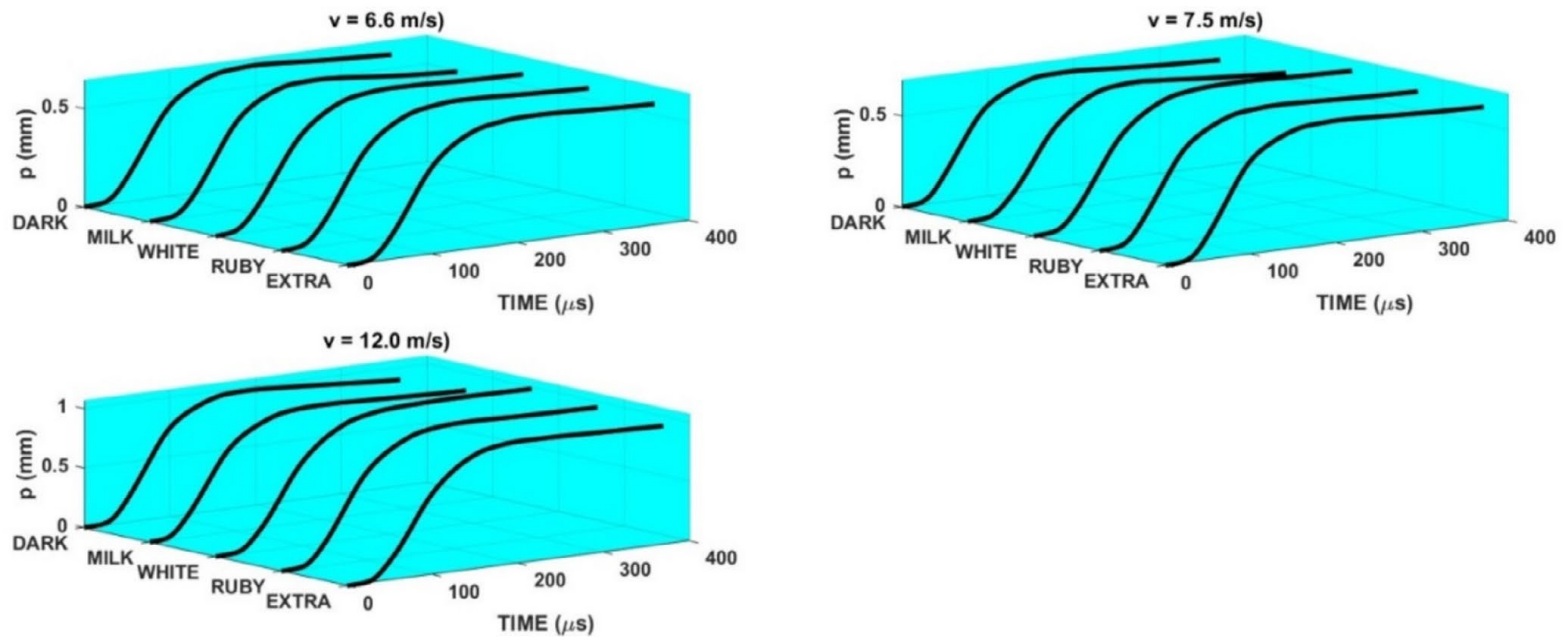
Displacements *p* of the chocolate specimens during the loading by the different stress pulses.

The loading of the specimen using the method displayed in Figure 2 is given by the striker impact velocity. Three levels of air pressure in the reservoir were used. Experiments were repeated five times under the same conditions (compressed air pressure). The striker impact velocities were 6.6 ± 0.23, 7.5 ± 0.26, and 12 ± 0.25 m/s. For the sake of simplicity, the values 6.6, 7.5, and 12 m/s were used for the description of results. In Figure 6, the examples of the loading stress pulses *σ*_*I*_(*t*) are given together with parameters of these pulses, that is, maximum of the stress pulse *σ*_*I*max_, impulse, and energy *w*_*I*_. The response of the specimen to these pulses is characterized by the reflected and transmitted stress pulses. Time histories of these pulses are displayed in Figure 7. The effect of the loading stress pulses on the transmitted stress pulses is shown in Figure 8. It is evident that the qualitative features of the transmitted stress pulses are nearly independent of the input stress pulse. The same is valid also for the time histories of the LR and specimen displacement *p*, see Figures 9 and 10.

**FIGURE 11:**
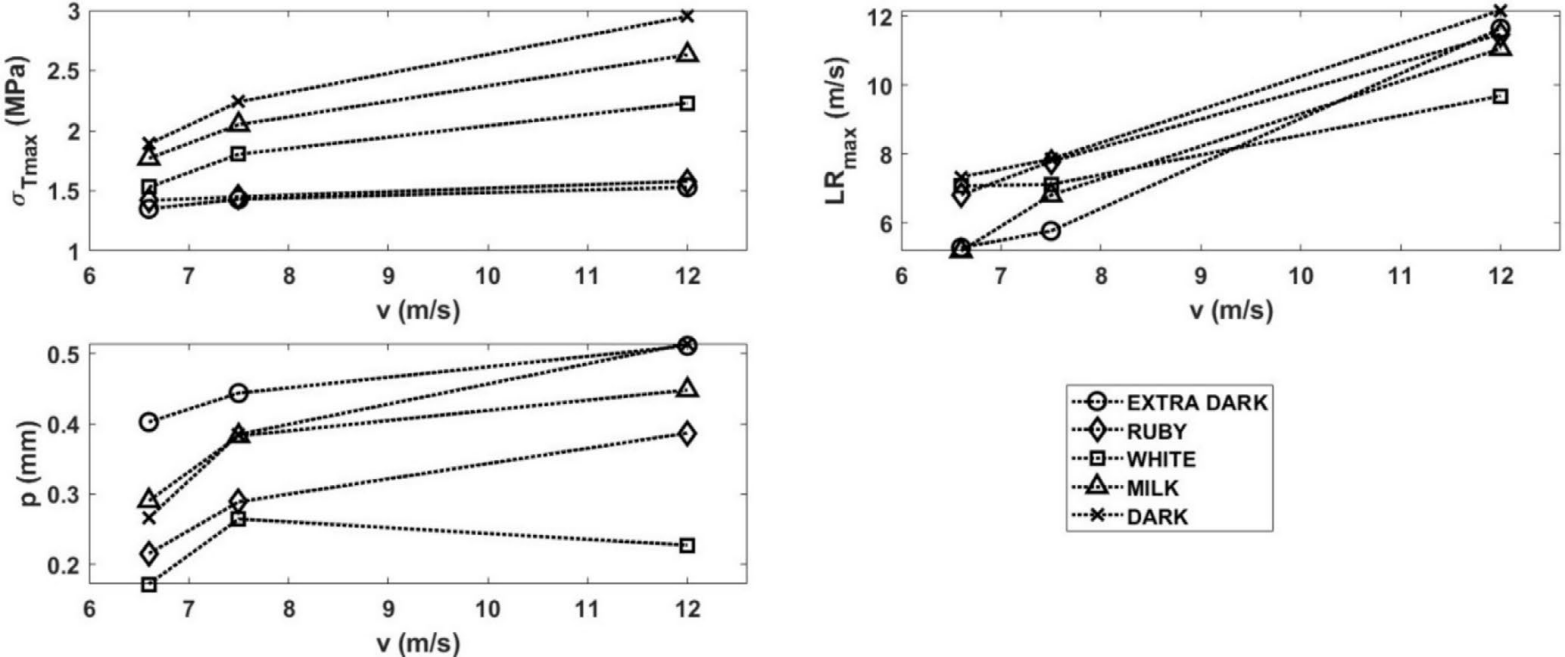
Parameters of the transmitted stress pulses.

Maximum (amplitude) of the transmitted stress pulse increases with the amplitude of the loading stress pulse, which is given by the striking velocity *v*, as shown in Figure 11. In this figure, the values of the LRs and the specimen displacement *p* at the time of achieving the amplitude maximum are also plotted.

If we take into consideration the stress pulse amplitude, there is no statistically significant difference (*p* < 0.05) between extra dark chocolate and ruby chocolates. The order of these amplitudes is the same as the order given by the static tensile strength; see Equation (10).

**FIGURE 12:**
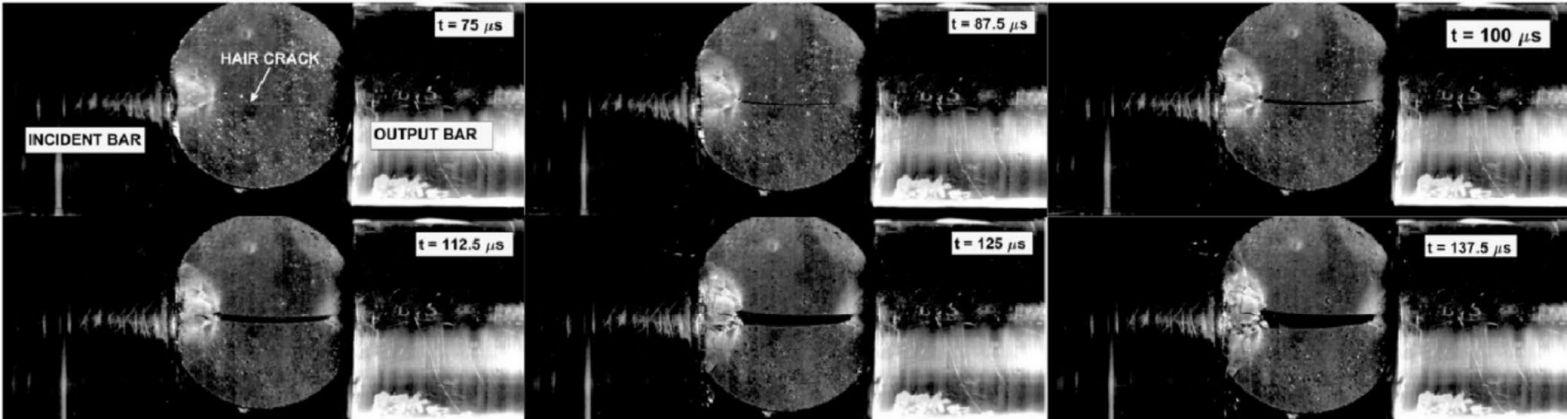
Development of fracture in the dark chocolate specimen loaded by SHPB technique. Striking velocity = 12 m/s.

**FIGURE 13:**
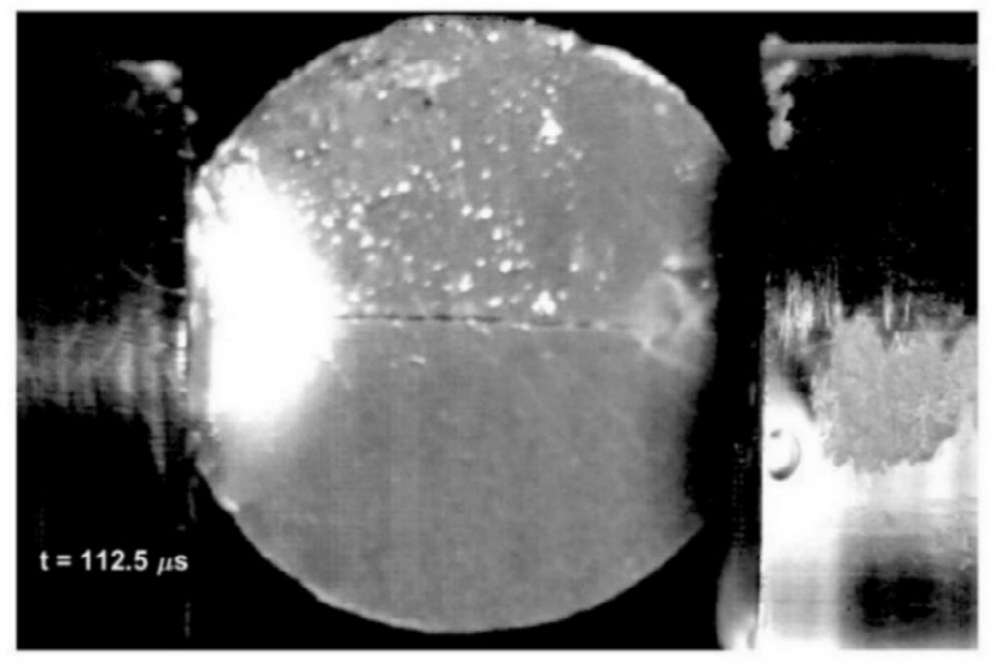
The area at the contact between the test bar and the milk chocolate specimen. Striking velocity = 12 m/s.

**FIGURE 14:**
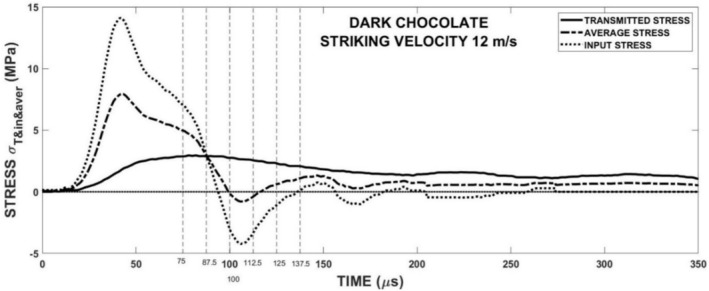
Stress histories in the specimen of the dark chocolate loaded by the stress pulse corresponding to the striking velocity = 12 m/s.

The behavior of the chocolate specimens during the loading was studied using a high‐speed camera. In Figure 12, the development of the specimen fracture damage is shown. The fracture starts in the form of a hair crack that is located nearly at the middle of the specimen. At the contact between the input bar and the specimen, the light area can be seen. A detailed view of this area is presented in Figure 13. In this area, the increase of the cocoa butter is detected. This increase in cocoa butter concentration is probably due to the stress concentration at the contact between the specimen and the input test bar, which allows some amount of cocoa butter to be released from the cocoa particles. This area is also reported at the contact between the specimen and the output bar. Its extent is significantly lower than that at the input bar–specimen contact. In Figure 14, the time histories of the transmitted stress pulse *σ*_*T*_(*t*) as well as the input stress:

(11) $$\sigma_{\text{input}}\left(t\right)=\sigma_{I}\left(t\right)+\sigma_{R}\left(t\right)$$

and the average stress:

(12) $$\sigma_{\text{average}}\left(t\right)=\frac{1}{2}\left[\sigma_{I}\left(t\right)+\sigma_{R}\left(t\right)+\sigma_{T}\left(t\right)\right]$$

are displayed. The *σ*_*I*_(*t*) is the incident stress pulse, *σ*_*R*_(*t*) is the reflected stress pulse, and *σ*_*T*_(*t*) is the part transmitted to the second bar as the stress pulse.

**FIGURE 15:**
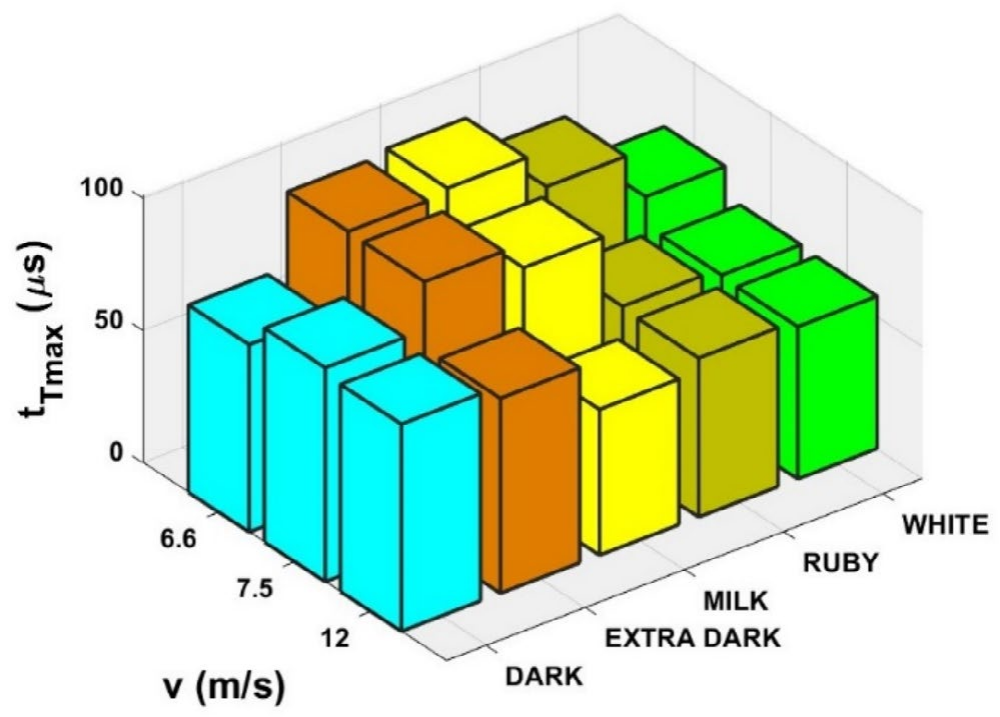
Times when the stress‐pulse amplitude is achieved.

**FIGURE 16:**
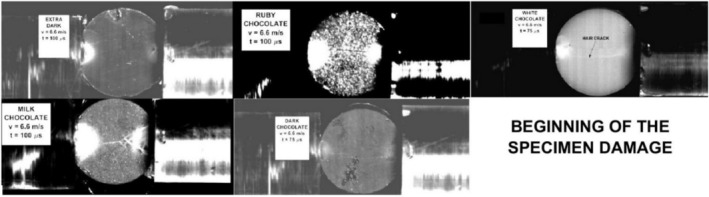
Start of the specimen damage. Striking velocity = 6.6 m/s.

The vertical lines in this figure correspond to the single frames in Figure 12. It is evident that the beginning of the specimen damage starts at the time which lies near the time when transmitted pulse amplitude is achieved. The times of achieving the transmitted stress amplitude are displayed in Figure 15. With the exception of the dark chocolate, this time decreases with the striking velocity, that is, with the input stress pulse amplitude. The initial fracture damage of the specimens is documented in Figure 16.

**FIGURE 17:**
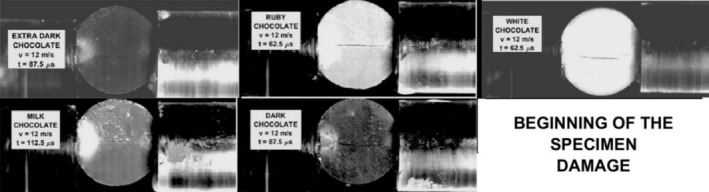
Start of the specimen damage. Striking velocity = 12 m/s.

It is evident that all reported damage occurs near the time when the transmitted pulse amplitude was achieved. The extent of the area of the cocoa butter concentration is highest for the milk chocolate and ruby chocolates. The same features exhibited chocolate specimens loaded by the more intensive stress pulses; see Figure 17.

**FIGURE 18:**
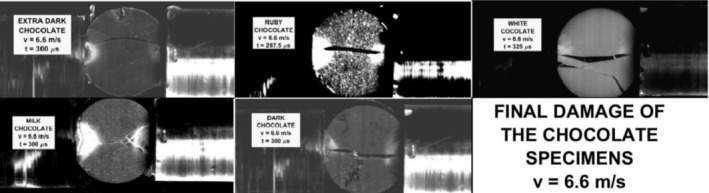
The final damage of the specimens loaded by the stress pulses corresponding to the striking velocity = 6.6 m/s.

**FIGURE 19:**
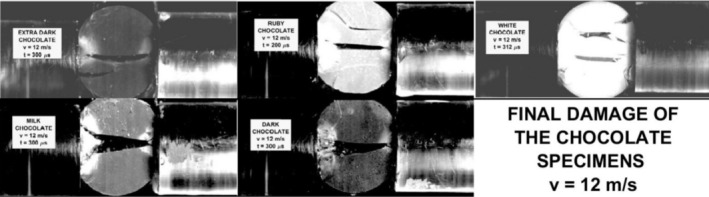
The final damage of the specimens loaded by the stress pulses corresponding to the striking velocity = 12 m/s.

The final damage of the tested chocolates is shown in Figure 18 for the lowest amplitudes of the input stress pulses and in Figure 19 for the highest stress pulse amplitudes.

The specimen damage is different for the different chocolates. With the exception of the milk chocolate and dark chocolates, one can observe a tendency for the multiple fracture damage of the specimens. The interpretation of obtained results on the fracture appearance needs some study of the fracture surfaces. In this paper, we focus on the quantitative evaluation of the tensile strength of the tested chocolates.

**FIGURE 20:**
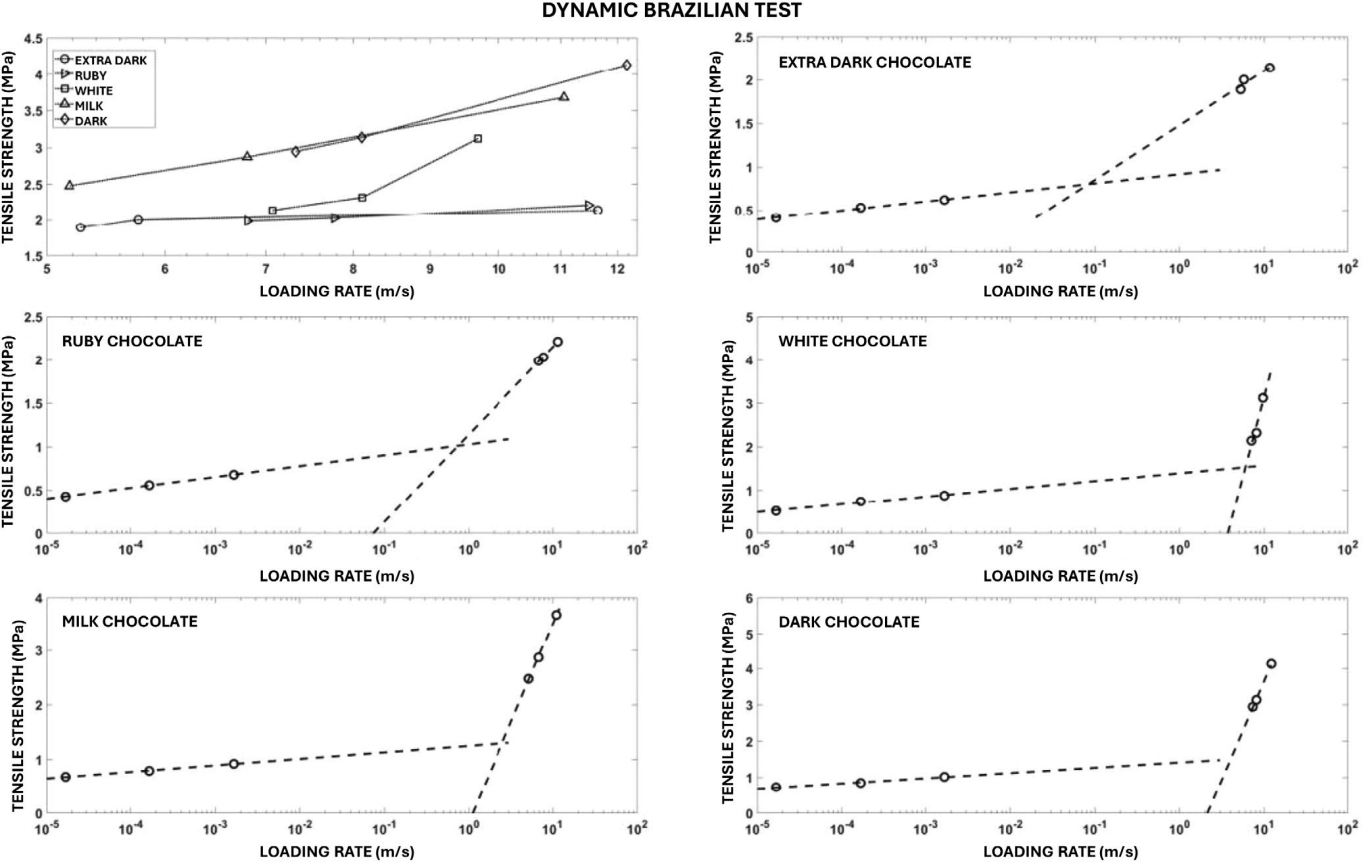
Effect of the loading rate on the dynamic tensile strength of the tested chocolates.

From Figure 14, it is evident that the stress in the specimen exhibits a significant gradient along the specimen length. The stress in the specimen is far from being homogeneous. Because the start of the fracture damage occurs near the transmitted stress pulse amplitude, the tensile strength of the specimen can be evaluated using Equation (6). This stress represents a minimum value of this strength. In Figure 20, the dependence of this strength on the LR is plotted.

It is evident that there is no or nearly no difference between the strengths of the extra dark chocolate and ruby chocolates as well as between milk chocolate and dark chocolates. The dynamic tensile strength of the white chocolate lies between. Dynamic tensile strength increases with the LRs. The explanation of the fracture behavior of different types of chocolate requires a detailed analysis of the chocolate. The main progress in solving this problem was made by Bikos et al. (2023) for some chocolate systems. As it is documented in Figure 20, the rate sensitivity of the tensile strength is significantly higher at dynamic loading than that reported during static loading. The effect of the LR on the tensile strength is qualitatively the same as the effect of the LR on the flow stress at the loading of the chocolate in compression, see Kumbár et al. (2023).

The mechanical properties of chocolate are clearly determined by its composition and production technology. The quality of the tempering process, the level of crystallization of the cocoa butter, and the stability of the cocoa fat crystal network formed are important factors (Afoakwa 2010). The hardness of chocolate decreases with the addition of the milk component due to the milk fat content, which changes the fatty acid composition in the continuous phase (Lapčíková et al. 2022). Chen et al. (2022) studied the effect of sugar and milk powder addition on the mechanical properties of chocolate. Mechanical properties, which are represented by Young's modulus and fracture toughness, correlate with textural properties such as hardness, elasticity and brittleness. The addition of sugar increased the value of stress impulse, Young's modulus, and fracture toughness of chocolate, whereras the opposite was true for the addition of milk powder. When both ingredients were equal, sugar played a more significant role. It is also confirmed, as reported by Liang and Hartel (2004), that the addition of milk powder at the expense of cocoa butter reduces the hardness of chocolate. The refractive properties of chocolates can also be significantly affected by the fat content. Zhao et al. (2018) found a decrease in both Young's modulus and fracture stress values of model chocolate bars as their fat content increased, which was due to an increase in the interaction between cocoa particles or cocoa particles and fat. In addition, the model chocolate bars with larger cocoa particle size had lower Young's modulus and fracture stress due to a lower proportion of free fat and a higher proportion of the empty matrix. Similarly, Afoakwa et al. (2009) pointed out that smaller particles and greater refinement of the individual components that make up the chocolate mass increase the tensile strength and hardness of the chocolate. And likewise, a higher proportion of fat acts as a lubricant, thereby reducing tensile strength and hardness.

## 4 Conclusions

The increase in the LR leads to the increase in the tensile strength of all tested chocolates. There are two regions of the tensile strength LR sensitivity. At LRs corresponding to the quasi‐static loading (typically achieved during loading at the conventional testing machines) there is a significant difference among different types of chocolates. The LR sensitivity of the tensile strength at higher LRs (typically for impact loading) is significantly higher. In these regions, there is nearly no difference in the tensile strength of ruby chocolate and extra dark chocolate and between milk chocolate and dark chocolates. The increase in tensile strength with LR is probably affected by the rheological properties of cocoa butter. The fracture of the specimen loaded by the high LRs starts in the middle of the specimen. At the contact between the specimen and bars, a region where cocoa butter increases. In order to obtain a more detailed view of this phenomenon, the numerical analysis of the stress development during the dynamic Brazilian test is desirable.

The obtained results can be used in industry for the correct processing of chocolate products and their transport. At the same time, the presented methods can be used to detect defects in chocolate products. There is also a certain correlation between the results presented and the sensory properties of chocolates. To monitor the quality of chocolate, it is certainly advisable for larger manufacturers to include at least some method of assessing the mechanical properties of chocolate in their quality testing. Standardized instruments for monitoring the textural properties of foods with variable LRs seem to be the most appropriate.

For future research, it would be useful to focus also on flavored/enriched chocolate masses, as our research focuses on pure basic types of chocolate.

## Author Contributions

**Šárka Nedomová:** methodology, investigation, validation, writing – review and editing. **Vojtěch Kumbár:** conceptualization, formal analysis, supervision, funding acquisition, project administration, writing – original draft. **Jan Trnka:** methodology, software, investigation, formal analysis, funding acquisition, writing – review and editing. **Veronika Šafránková:** data curation, investigation, resources, writing – review and editing. **Renáta Dufková:** data curation, investigation, resources. **Jiří Votava:** conceptualization, methodology, data curation. **Luděk Hřivna:** data curation, supervision, resources, writing – review and editing. **Jaroslav Buchar:** conceptualization, methodology, validation, visualization, writing – original draft.

## Ethics Statement

The authors have nothing to report.

## Consent

Written informed consent was obtained from all study participants.

## Conflicts of Interest

The authors declare no conflicts of interest.

## Data Availability

The data that support the findings of this study are available from the corresponding author upon reasonable request.

## References

[jtxs70021-bib-0001] Afoakwa, E. O. 2010. Chocolate Science and Technology. Willey‐Blackwell.

[jtxs70021-bib-0002] Afoakwa, E. O. , A. Paterson , M. Fowler , and J. Vieira . 2009. “Microstructure and Mechanical Properties Related to Particle Size Distribution and Composition in Dark Chocolate.” International Journal of Food Science & Technology 44, no. 1: 111–119. 10.1111/j.1365-2621.2007.01677.x.

[jtxs70021-bib-0003] Beckett, S. T. 2008. The Science of Chocolate. 2nd ed, 240. Royal Society of Chemistry. 10.1039/9781847558053.

[jtxs70021-bib-0004] Bikos, D. , G. Samaras , P. Cann , et al. 2021. “Effect of Micro‐Aeration on the Mechanical Behaviour of Chocolates and Implications for Oral Processing.” Food & Function 12, no. 11: 4864–4886. 10.1039/d1fo00045d. 33969364

[jtxs70021-bib-0005] Bikos, D. , G. Samaras , P. Cann , et al. 2023. “Destructive and Non‐Destructive Mechanical Characterisation of Chocolate With Different Levels of Porosity Under Various Modes of Deformation.” Polymers and Biopolymers 58: 5104–5127. 10.1007/s10853-023-08324-7.

[jtxs70021-bib-0006] Chen, J. 2009. “Food Oral Processing—A Review.” Food Hydrocolloids 23: 1–25. 10.1016/j.foodhyd.2007.11.013.

[jtxs70021-bib-0007] Chen, W. , and B. Song . 2011. Split Hopkinson (Kolsky) Bar. Mechanical Engineering Series. Springer. 10.1007/978-1-4419-7982-7.

[jtxs70021-bib-0008] Chen, Y. Y. , X. Y. Zhou , S. H. Qian , and J. H. Yu . 2022. “Effect of Sugar and Milk Powder Addition on the Mechanical Properties and Texture of Chocolate.” Journal of Oleo Science 71, no. 11: 1577–1589. 10.5650/jos.ess22148. 36198582

[jtxs70021-bib-0009] Dai, F. , S. Huang , K. Xia , and Z. Tan . 2010. “Some Fundamental Issues in Dynamic Compression and Tension Tests of Rocks Using Split Hopkinson Pressure Bar.” Rock Mechanics and Rock Engineering 43, no. 6: 657–666. 10.1007/s00603-010-0091-8.

[jtxs70021-bib-0010] Kim, E. H. J. , V. K. Corrigan , A. J. Wilson , I. R. Waters , D. I. Hedderley , and M. P. Morgenstern . 2012. “Fundamental Fracture Properties Associated With Sensory Hardness of Brittle Solid Foods.” Journal of Texture Studies 43, no. 1: 49–62. 10.1111/j.1745-4603.2011.00316.x.

[jtxs70021-bib-0011] Kumbár, V. , V. Kouřilová , R. Dufková , J. Votava , and L. Hřivna . 2021. “Rheological and Pipe Flow Properties of Chocolate Masses at Different Temperatures.” Food 10, no. 11: 2519. 10.3390/foods10112519. PMC8621481 34828800

[jtxs70021-bib-0012] Kumbár, V. , J. Trnka , V. Kouřilová , et al. 2024. “Stress Wave Attenuation in Chocolate.” Journal of Food Engineering 347: 112037. 10.1016/j.jfoodeng.2024.112037.

[jtxs70021-bib-0013] Kumbár, V. , J. Trnka , V. Kouřilová , et al. 2023. “High Strain Rate Behaviour of Different Types of Chocolate.” Journal of Food Engineering 346: 111438. 10.1016/j.jfoodeng.2023.111438.

[jtxs70021-bib-0014] Lapčíková, B. , L. Lapčík , R. Salek , T. Valenta , and E. Lorencová . 2022. “Physical Characterization of the Milk Chocolate Using Whey Powder.” LWT—Food Science and Technology 154: 112669. 10.1016/j.lwt.2021.112669.

[jtxs70021-bib-0015] Li, D. , and L. N. Y. Wong . 2013. “The Brazilian Disc Test for Rock Mechanics Applications: Review and New Insights.” Rock Mechanics and Rock Engineering 46, no. 2: 269–287. 10.1007/s00603-012-0257-7.

[jtxs70021-bib-0016] Liang, B. , and R. W. Hartel . 2004. “Effects of Milk Powders in Milk Chocolate.” Journal of Dairy Science 87, no. 1: 20–31. 10.3168/jds.S0022-0302(04)73137-9. 14765806

[jtxs70021-bib-0017] Nedomová, Š. , J. Trnka , and J. Buchar . 2013. “Tensile Strength of the Dark Chocolate.” Acta Technologica Agriculturae 16, no. 3: 71–73. 10.2478/ata-2013-0018.

[jtxs70021-bib-0018] Nedomová, Š. , J. Trnka , V. Kouřilová , et al. 2023. “Acoustic Properties and Low Strain Rate Behavior of Different Types of Chocolate.” International Journal of Food Properties 26, no. 1: 842–854. 10.1080/10942912.2023.2189087.

[jtxs70021-bib-0019] Rahner, C. , H. A. Al‐Qureshi , D. Stainer , D. Hotza , and M. C. Fredel . 2014. “Numerical Evaluation of a Light‐Gas Gun Facility for Impact Test.” Modelling and Simulation in Engineering 2014: 501434. 10.1155/2014/501434.

[jtxs70021-bib-0020] Sheikh, M. Z. , Z. Wang , B. Du , et al. 2019. “Static and Dynamic Brazilian Disk Tests for Mechanical Characterization of Annealed and Chemically Strengthened Glass.” Ceramics International 45, no. 6: 7931–7944. 10.1016/j.ceramint.2019.01.106.

[jtxs70021-bib-0021] Tremeac, B. , M. Hayert , and A. Le‐Bail . 2008. “Mechanical Properties of Tylose Gel and Chocolate in the Freezing Range.” International Journal of Refrigeration 31, no. 5: 867–873. 10.1016/j.ijrefrig.2007.10.005.

[jtxs70021-bib-0022] Wang, J. , and J. Tao . 2022. “Determination of Tensile Strength at Crack Initiation in Dynamic Brazilian Disc Test for Concrete‐Like Materials.” Buildings 12, no. 6: 797. 10.3390/buildings12060797.

[jtxs70021-bib-0023] Yu, R. C. , G. Ruiz , and A. Pandolfi . 2004. “Numerical Investigation of the Dynamic Behaviour of Advanced Ceramics.” Engineering Fracture Mechanics 71, no. 4–6: 897–911. 10.1016/S0013-7944(03)00016-X.

[jtxs70021-bib-0024] Zhao, H. , B. Li , and B. J. James . 2018. “Structure‐Fracture Relationships in Chocolate Systems.” LWT—Food Science and Technology 96: 281–287. 10.1016/j.lwt.2018.05.045.

